# Sternotomy after retrosternal esophagogastric anastomotic disruption: a case report

**DOI:** 10.1186/1757-1626-2-7523

**Published:** 2009-05-08

**Authors:** Hassan Jamal-Eddine, Murugan Sukumar, Nael Al-Sarraf

**Affiliations:** Department of Thoracic Surgery, Chest Disease HospitalAl-Jabriah, P.O. Box. 718Kuwait

## Abstract

Disruption of cervical esophagogastric anastomosis after retrosternal stomach transposition remains a dangerous complication. We report a case of cervical gastric disruption after retrosternal gastric transposition in a 36-year-old man that required sternotomy for reanastomosis. After sternotomy, gastric mobilization was possible, in order to gain sufficient length for a new cervical esophagogastric anastomosis.

## Introduction

Various approaches have been described in dealing with disruption of cervical esophagogastric anastomosis. Here we report a complicated case of Boerhaave’s syndrome successfully treated by using median sternotomy approach thereby avoiding more dangerous options.

## Case presentation

**Figure 1.**
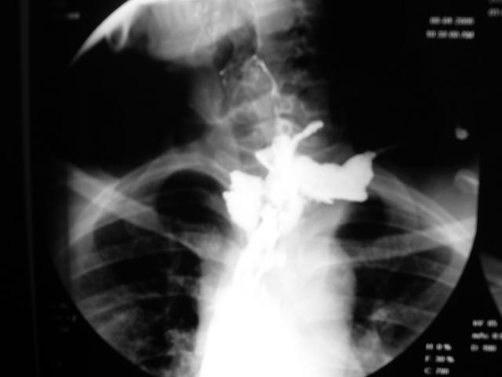
Esophagogram showing complete disruption of the cervical esophagogastric anastomosis.

A 36-year-old Indian man was hospitalized 72 hours after forceful vomiting followed by the onset of chest pain, dyspnoea and fever. The patient had no previous medical history and no family history of relevance. He worked as construction worker. He was non-smoker and consumed an average of 40 unit of alcohol per week. His height was 170 cm and his weight was 64 kg. Initial chest radiography showed left-sided hydropneumothorax with mediastinal emphysema. Computed tomography of the chest with contrast showed spontaneous esophageal perforation (Boerhaave’s syndrome) in the left lower third of thoracic esophagus. Patient underwent emergency direct surgical repair with pleural flap reinforcement (first operation). Subsequently he developed an esophageal leak with mediastinitis and empyema, followed by a septic shock. Esophagectomy with cervical esophagostomy, gastrostomy and feeding jejunostomy was done (second operation) with a preparation for esophageal reconstruction at a later stage. Six weeks later, a retrosternal gastric transposition with CEGA was performed using circular stapler (third operation). Transmediastinal gastric transposition was found to be difficult because of the two previous surgeries and inflammatory process. Five days later, the patient developed discharge from the neck incision which required bedside drainage and packing. Contrast study revealed a major disruption of the cervical anastomosis (Figure 1).

**Figure 2.**
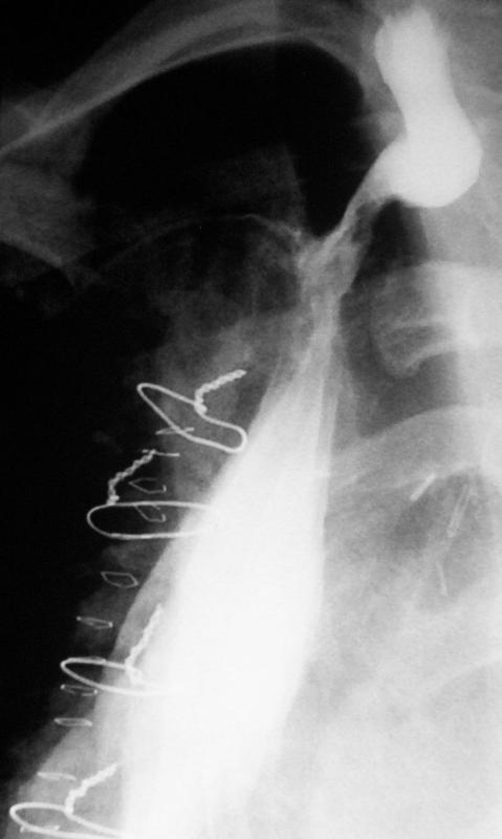
Postoperative esophagogram showing preserved esophagogastric continuity.

Patient underwent exploration which revealed a necrosis of the gastric tip, resulting in complete anastomotic disruption. Complete median sternotomy was done with extra care not to injure the retrosternal gastric conduit, followed with tedious blunt dissection of the stomach that was firmly adherent to the sternum anteriorly and mediastinum posteriorly. Finally, it was freed with maximum possible mobilization, in order to gain a sufficient length. After generous excision of the necrotic gastric tip and the edge of cervical esophagus, a new CEGA was performed, using manual anastomosis with interrupted 4-0 vicryl (forth operation). Patient made steady postoperative recovery with no signs of sterna infection or mediastinal collection. On the 10th postoperative day esophagogram with gastrograffin showed a patent and competent anastomosis (Figure 2). Patient was discharged in a stable condition and remained well six months later.

## Discussion

The stomach is the optimal organ for esophageal replacement for both benign and malignant diseases [1], while esophageal resection is necessary to eliminate the source of intrathoracic sepsis [2]. After esophageal resection and diversion, staged reconstruction is the only rational option for these patients [3]. However, in the presence of previous unsuccessful surgeries, reconstruction of the esophagus could be quite challenging [4]. With obliteration of the posterior mediastinum due to prior surgery, the retrosternal approach is the better option [1,5], but it is associated with a higher incidence of anastomotic leak, compared to gastric reconstructions through posterior mediastinum [4,5]. Restoration of swallowing after CEGA has been associated with more frequent leaks than if anastomosis was located in the thoracic cavity [5-7]. Possible explanations include tension, extrinsic compression, the mesothelial environment in the neck, surgical experience and technique, as well as good gastric conduit vascularity [5,6]. High index of clinical suspicion and contrast examination are the keys for early diagnosis [5,8]. Leaks occurring in the early postoperative period are usually due to conduit necrosis with a significant anastomotic disruption [5-7]. Early optimal surgical treatment after occurrence of this complication is mandatory. Cervical leak management include opening the cervical incision and drainage [5-7]. However, with a large leak, local care is inadequate to control the problem [5,6]. Hence, aggressive surgical approach, in the form of resection of the necrotic part, mediastinal debridement followed by cervical reanastomosis, can preserve the gastroesophageal continuity, thus avoiding the possibility of another complex surgery [2]. Potential surgical options that could have been attempted include either positioning the esophageal replacement in the posterior mediastinal bed (which proved impossible due to multiple previous operations) or using the retrosternal approach (with or without resection of the medial part of clavicle and sternoclavicular joint). The advantage of resecting such a joint is to enlarge the anterior opening into the superior mediastinum thus preventing impingement by posterior prominence of clavicular head [1,4]. The disadvantage of resecting the sternoclavicular joint includes early shoulder drop with potential compression on the stretched subclavian vein over the first rib and swelling of ipsilateral arm [1]. Using retrosternal approach, we were able to fashion our anastomosis without resecting the sternoclavicular joint, thereby preventing any additional postoperative morbidity. In the presence of multiple previous surgeries, any approach could be problematic, particularly with previous retrosternal gastric transposition. In our case, the only option left was to take down the stomach back to abdomen (after dissection from adhesions, partial resection and debridement) and prepare the patient for the next (fifth operation) stage - colonoplasty or free jejunal graft.

The ultimate goal in CEGA is to restore the function of swallowing as close to normal as possible, with minimal morbidity and mortality. Using median sternotomy we were able to mobilize the adherent stomach enough to resect the necrotic part of the previous anastomosis and perform a new esophago-gastric anastomosis and establish gastrointestinal continuity without complication. It proved to be the most appropriate strategy, thus avoiding other, more complicated and hazardous surgical alternatives in benign esophageal disease.

## Conclusion

The use of median sternotomy can be safely attempted in complicated cases of CEGA disruption with successful outcome avoiding the need for more hazardous surgical procedures.

## Abbreviation

CEGA: Cervical esophagogastric anastomosis
